# Supplementary figures and images for: Evolution of the interactions between GII.4 noroviruses and histo-blood group antigens: Insights from experimental and computational studies
Source: PLoS Pathog. 2021 Jul 12;17(7):e1009745. doi: 10.1371/journal.ppat.1009745 (PMC8297928; doi:10.1371/journal.ppat.1009745)

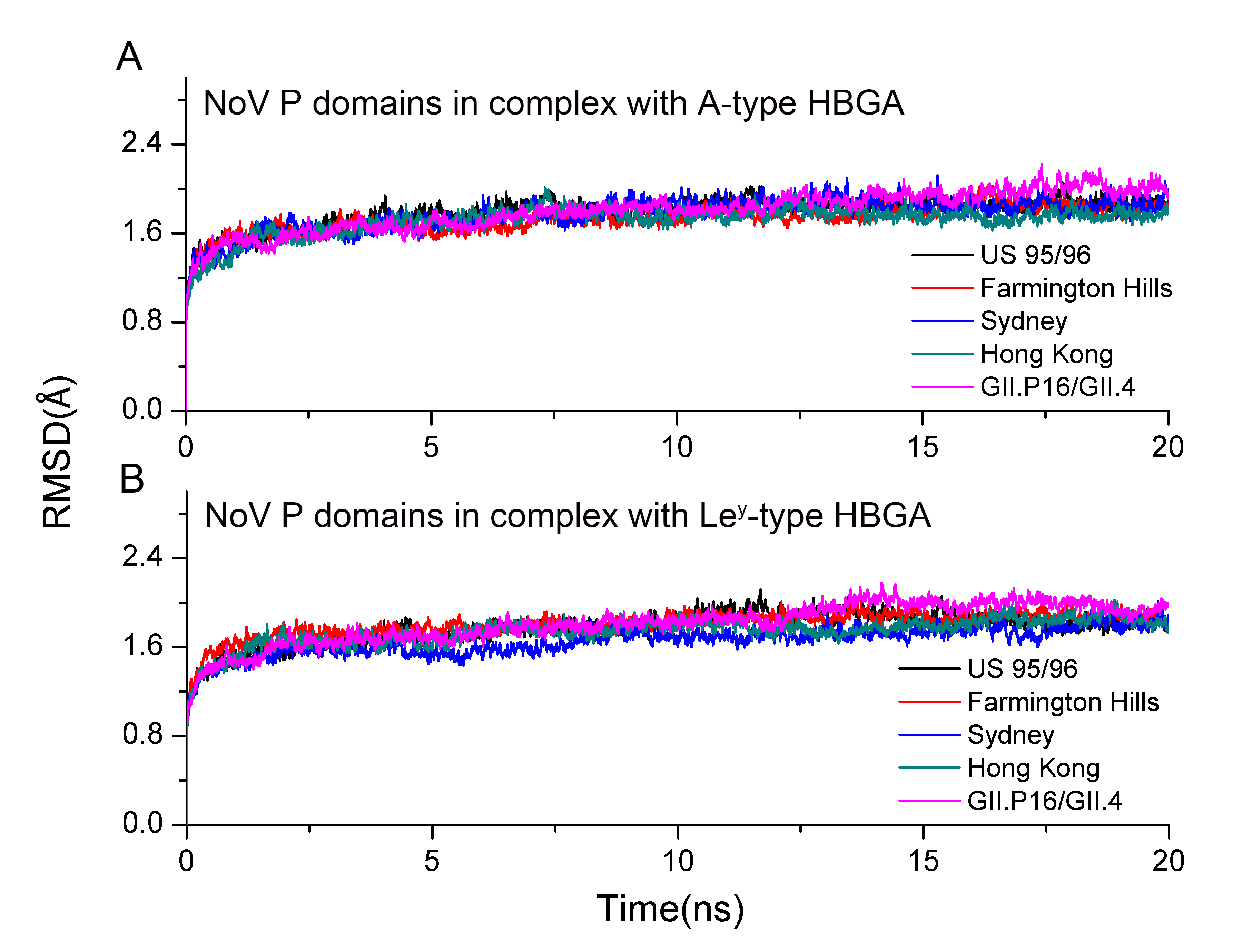

Supplement: S1 Fig — (A) The RMSD plots for the P domains of the US95/96 (black), Farmington Hills (red), Sydney (blue), Hong Kong (green) and GII.P16/GII.4 (purple) strains, respectively, in complex with type A HBGA. (B) The RMSD plots for the P domains of the US95/96 (black), Farmington Hills (red), Sydney (blue), Hong Kong (green) and GII.P16/GII.4 (purple) strains, respectively, in complex with type Ley HBGA. (TIF) [file ppat.1009745.s001.tif]

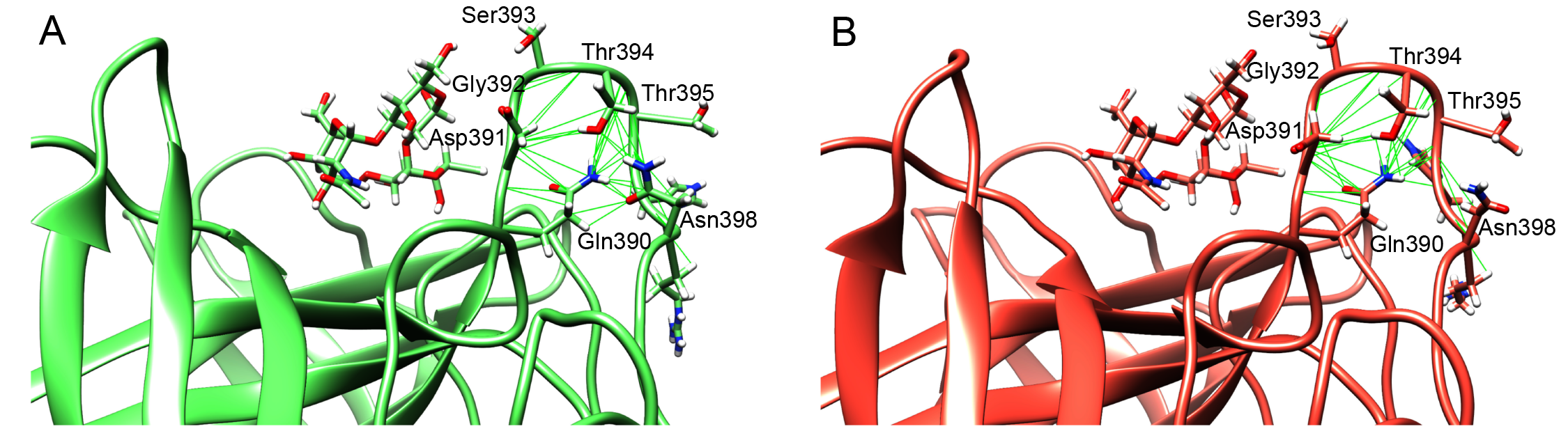

Supplement: S2 Fig — (A) The residue contacts in loop-2 for the Hong Kong strain. (B) The residue contacts in loop-2 for the GII.P16/GII.4 strain. (TIF) [file ppat.1009745.s002.tif]

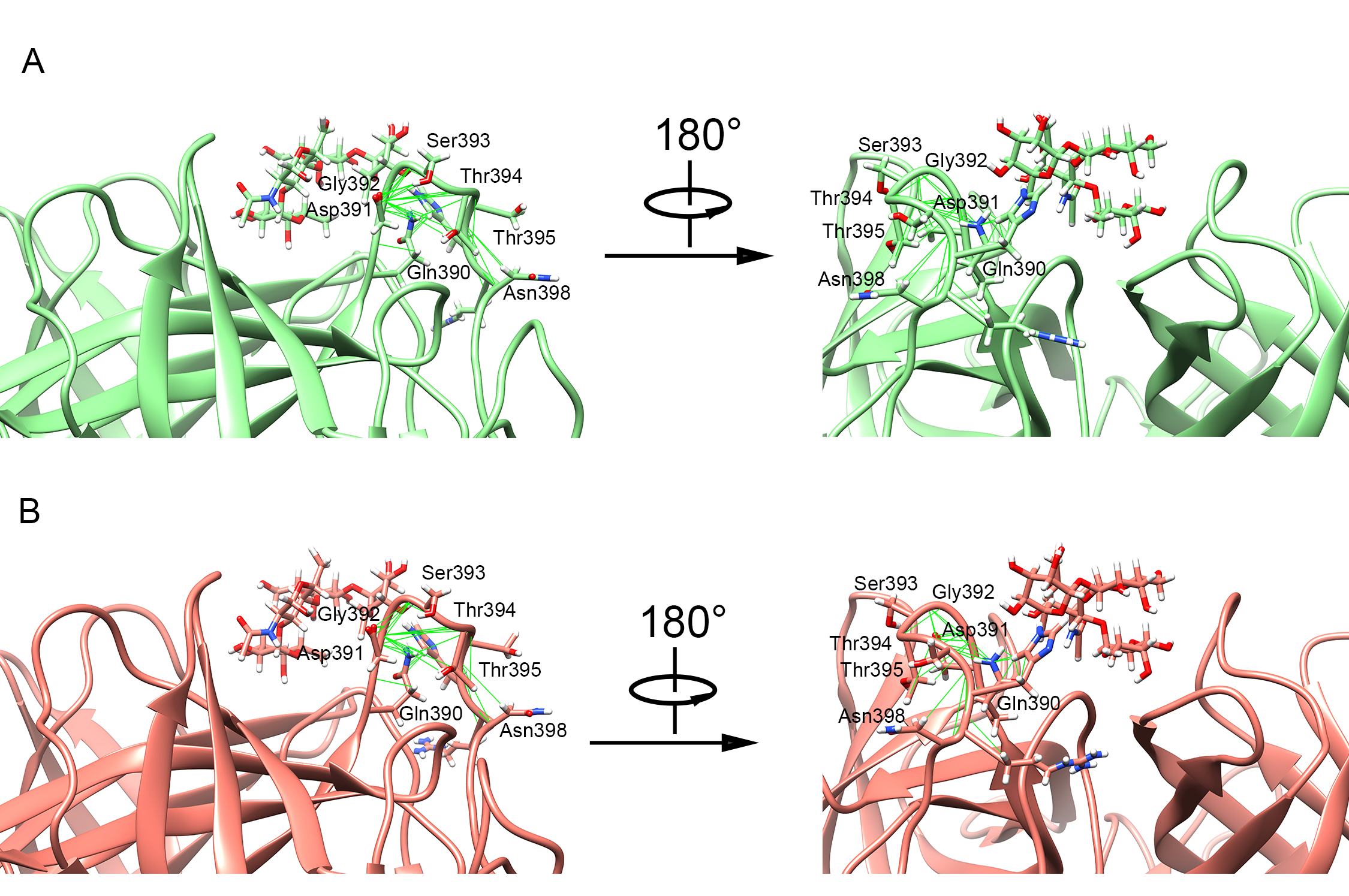

Supplement: S3 Fig — (A) The residue contacts in loop-2 for the Hong Kong strain. (B) The residue contacts in loop-2 for the GII.P16/GII.4 strain. (TIF) [file ppat.1009745.s003.tif]
