# Supplementary material for: Evolution of the interactions between GII.4 noroviruses and histo-blood group antigens: Insights from experimental and computational studies
Source: PLoS Pathog. 2021 Jul 12;17(7):e1009745. doi: 10.1371/journal.ppat.1009745 (PMC8297928; doi:10.1371/journal.ppat.1009745)
Supplement: Attachment — Submitted filename: Response to reviewers.docx [file ppat.1009745.s005.docx]

**Manuscript No.:** **PPATHOGENS-D-20-02500 - [EMID:8e54257b7226a3b4]**

**Title:** Evolution of the interactions between GII.4 noroviruses and histo-blood group antigens: Insights from experimental and computational studies 
**Author(s):** Yu Liang, Wei Bu Wang, Jing Zhang, Jun Wei Hou, Fang Tang, Xue Feng Zhang, Li Fang Du, Ji Guo Su, Qi Ming Li

Dear Editors and Reviewers,

We would like to thank the editors and the reviewers for the dedicated efforts in reviewing our manuscript.

We have carefully studied the valuable comments of the editors and the reviewers, and revised our manuscript according to the suggestions. All the revised portions are marked in red in the revised manuscript. The responses to reviewer’s comments and the corresponding main revisions in the paper are as following:

**Responses to Reviewer’s Comments:**

This study is an additional analysis of the known factor that is associated with susceptibility and infection of humans and noroviruses. They use yeast expression to prepare the capsids of several strains from a recent common lineage (GII.4) of human noroviruses that has become widespread around the world. They perform binding studies to the different blood group glycans, showing increasing binding over time of evolution of the viruses. They model the docking of the glycans into the structures of the viruses based on the sequences and known structures of related viruses binding to the different blood group antigens. The data is similar to previous studies, but adds additional approaches as well as details from the more recent viruses. That data supports the general connection of the affinity of binding to the increased spread of the viruses, but there is no direct connection back to the disease in vivo, and data is mostly suggestive. The manuscript is clear and well presented, and the data appear to be fine as far as they go.

Reviewer Comments (if any, and for reference):

Reviewer's Responses to Questions

**Part I - Summary**
Please use this section to discuss strengths/weaknesses of study, novelty/significance, general execution and scholarship.

Reviewer #1: In this article, the authors study the interactions between different types of histo-blood antigens (HBGAs) and virus-like particles of nine representative genotype II.4 strains of Norovirus. This work aims to understand binding affinity evolution at the structural (atomic) level. Indeed, the biding activity of HBGAs increases along the evolution of genotype II.4. The study combines both experimental and computational methods. In particular, the authors used molecular dynamics and molecular mechanic/generalized born surface area calculations to interpret enzyme linked immunosorbent assays. This allows the authors to interpret mutations consequences for binding affinity.
It is a very interesting work. The results are clear and well described. The methods are rigorously applied with correct interpretation.
This work is suitable for the audience of Plos Pathogens.

**Part II – Major Issues: Key Experiments Required for Acceptance**
Please use this section to detail the key new experiments or modifications of existing experiments that should beabsolutely required to validate study conclusions.

Generally, there should be no more than 3 such required experiments or major modifications for a "Major Revision" recommendation. If more than 3 experiments are necessary to validate the study conclusions, then you are encouraged to recommend "Reject".

Reviewer #1: (No Response)

**Part III – Minor Issues: Editorial and Data Presentation Modifications**
Please use this section for editorial suggestions as well as relatively minor modifications of existing data that would enhance clarity.

Reviewer #1: Minor point: the authors indicated p25 line 531 that 100 snapshots were used to compute the binding free energy for each simulation. However, in figure 4B, the curves presented do not take account of the statistics. I guess the means are plotted but the authors should show error bars.
It would also be informative to present the RMSD for each molecular dynamic simulations in the SI.

**Response:** Thank you so much for your valuable suggestions. In our binding free energy calculations, both the mean value and the standard error of the mean (SEM) can be obtained by using 100 frames from the MD simulation. In Figure 4b, only the mean values were displayed. According to your suggestion, in the revised manuscript, the SEM values were also presented as error bars in the figure.

According to your suggestions, the time-evolution of RMSD for the MD simulation of each studied complex was presented in S1 Fig., and a description of the RMSD plots was also added in the revised manuscript (see line 239-243 on page 11 highlighted in red).

PLOS authors have the option to publish the peer review history of their article ([what does this mean?](https://journals.plos.org/plospathogens/s/editorial-and-peer-review-process#loc-peer-review-history)). If published, this will include your full peer review and any attached files.


If you choose “no”, your identity will remain anonymous but your review may still be made public.


**Do you want your identity to be public for this peer review?** For information about this choice, including consent withdrawal, please see our [Privacy Policy](https://www.plos.org/privacy-policy).

Reviewer #1: No

Figure Files:

While revising your submission, please upload your figure files to the Preflight Analysis and Conversion Engine (PACE) digital diagnostic tool, https://pacev2.apexcovantage.com. PACE helps ensure that figures meet PLOS requirements. To use PACE, you must first register as a user. Then, login and navigate to the UPLOAD tab, where you will find detailed instructions on how to use the tool. If you encounter any issues or have any questions when using PACE, please email us at figures@plos.org.

Data Requirements:

Please note that, as a condition of publication, PLOS' data policy requires that you make available all data used to draw the conclusions outlined in your manuscript. Data must be deposited in an appropriate repository, included within the body of the manuscript, or uploaded as supporting information. This includes all numerical values that were used to generate graphs, histograms etc.. For an example see here:<http://www.plosbiology.org/article/info%3Adoi%2F10.1371%2Fjournal.pbio.1001908#s5>.

**Response:** The numerical data that were used to generate all figures are included in S1 Data in the Supporting information of the manuscript.
